# Impact of Dermatologic Screening and Methods on Breslow Thickness in Melanoma: A Retrospective Cohort Study

**DOI:** 10.3390/cancers18030461

**Published:** 2026-01-30

**Authors:** Katharina Wunderlich, Apolline Potiez, Carmen Orte Cano, Joanna Bouchat, Nancy Van Damme, Mariano Suppa, Jonathan M. White, Hassane Njimi, Elizabeth Van Eycken, Véronique Del Marmol

**Affiliations:** 1 Department of Dermatology, Hôpital Erasme, Université Libre de Bruxelles, 1070 Brussels, Belgium; 2 Belgian Cancer Registry, 1210 Brussels, Belgiumnancy.vandamme@kankerregister.org (N.V.D.); elizabeth.vaneycken@kankerregister.org (E.V.E.); 3 Department of Dermatology, Institute Jules Bordet, Université Libre de Bruxelles, 1070 Brussels, Belgium

**Keywords:** melanoma, skin cancer screening, dermoscopy, digital mole mapping, Breslow thickness

## Abstract

Background/Objectives: Melanoma is the most lethal cutaneous neoplasm, with Breslow thickness being a key prognostic factor. This retrospective cohort study aimed to assess the impact of screening frequency and diagnostic methods on tumour stage at diagnosis and to explore implications for risk-adapted strategies. Methods: Between 2017 and 2024, 475 cases of melanoma were diagnosed in 397 patients. Screening frequency, diagnostic method, and patient risk were analyzed in relation to tumour stage. Results: Compared with first-visit cases, patients who underwent screening within two years prior to diagnosis were more often diagnosed with melanoma in situ (32.6% vs. 44–51%; *p* < 0.05) and had thinner invasive tumours (0.68–0.73 mm vs. 1.8 mm; *p* ≤ 0.001), though no differences were seen between screening frequencies. Full-body examination was associated with more in situ melanomas (46% vs. 34%; *p* = 0.016) and thinner invasive tumours (0.92 vs. 2.05 mm; *p* = 0.2) compared with lesion-directed screening, but this effect disappeared after excluding first-visit cases. Invasive melanomas diagnosed by mole mapping were significantly thinner than by dermoscopy (0.55 vs. 1.07; *p* = 0.035). In high-risk patients, tumour thickness decreased with more frequent visits (0.905 mm without screening vs. 0.40–0.55 mm with ≥1 visit; *p* = 0.001). Moreover, mole mapping identified thinner melanomas in the high-risk group compared with dermoscopy (0.47 vs. 0.60 mm; *p* = 0.02). Conclusions: Screening is associated with thinner melanomas and more in situ diagnoses. Digital mole mapping offers additional benefits, with high-risk patients profiting most, while low-risk individuals could be managed with less resource-intensive approaches. These findings support risk-adapted screening strategies focusing on intensive, digitally supported modalities for high-risk groups.

## Simple Summary

This retrospective cohort study of 475 cases of melanoma diagnosed at Erasme University Hospital (2017–2024) shows that dermatological screening is associated with thinner tumours and more melanoma in situ compared with first-visit diagnoses. Full-body examination detected earlier-stage disease than lesion-directed screening, while digital mole mapping identified significantly thinner invasive melanomas than dermoscopy, even after excluding first-visit cases. Screening and digital mole mapping had no significant benefit in low-risk patients. However, in high-risk patients, tumour thickness decreased with screening and digital mole mapping. These findings support risk-adapted screening strategies, with intensified, digitally supported follow-up recommended for high-risk groups.

## 1. Introduction

Melanoma is a malignant tumour that arises from melanocytes and primarily involves the skin. It is the most lethal cutaneous neoplasm, accounting for approximately 90% of skin cancer-related deaths. Over the last three decades, melanoma mortality has shown a favourable pattern in most European countries, the USA, Australia, and several Latin American countries. This is a trend attributable to prevention, earlier diagnosis, and significant advances in targeted therapies. This has occurred alongside a steady rise in the global incidence of malignant melanoma over the past five decades—a trend closely linked to lifestyle factors, particularly ultraviolet (UV) radiation exposure. UV radiation is estimated to be responsible for over 90% of all melanoma cases. In addition, improvements in screening methods and increasing frequency of skin check-ups have contributed to greater detection, raising concerns about potential overdiagnosis [1,2,3,4,5].

Several well-established risk factors have been associated with the development of cutaneous melanoma. Among them is the number of melanocytic naevi: individuals with a high count of common naevi (101–120) exhibit an approximately sevenfold increased risk of melanoma compared to those with few naevi (0–15). Likewise, the presence of five or more atypical naevi confers a sixfold higher risk relative to individuals without atypical naevi. Overall, melanoma arises from a multifactorial interplay of intrinsic factors—including fair phototype, light hair and eye colour, freckles, high or atypical nevus count, male sex, personal and family history of melanoma, and germline mutations in genes such as CDKN2A or MC1R—and extrinsic exposures such as alcohol consumption, immunosuppression, and especially intermittent solar or artificial UV radiation and childhood sunburns [6,7,8,9,10,11,12,13,14,15,16,17,18,19].

Approximately 90% of melanomas are diagnosed as primary tumours without evidence of metastasis at the time of diagnosis. The tumour-specific 10-year survival rate for these cases ranges from 85% to 95%. The most important histopathological prognostic factors for primary melanoma include the vertical tumour thickness (Breslow thickness), the presence of histologically defined ulceration, and the mitotic rate. Breslow thickness remains a crucial prognostic factor in melanoma [2,3].

Large-scale, population-based data from Australia (1982–2014) demonstrate that long-term melanoma-specific mortality is strongly associated with Breslow thickness. At 30 years, the cumulative incidence of melanoma-related death was 7.1% for T1 tumours (≤1.0 mm), 21.6% for T2 (1.1–2.0 mm), 34.2% for T3 (2.1–4.0 mm), and 44.3% for T4 (>4.0 mm) melanomas. Correspondingly, 30-year melanoma-specific survival declined from 94.9% in the T1 to 53.8% in the T4 categories. These findings highlight that, although thin melanomas are numerically responsible for a substantial proportion of deaths due to their high incidence, Breslow thickness is a crucial prognostic factor [20].

Enhanced screening efforts have likely contributed to the increased detection of melanoma in situ and early-stage thin melanomas, which are associated with a more favourable prognosis [21]. However, screening the general population also comes with difficulties like overdiagnosis, a high number needed to screen (>8000) to avoid one melanoma death, and limited access to dermatological resources [22]. The current European guidelines recommend that the clinical diagnosis of melanoma is based on several key components: a total-body visual examination to detect lesions exhibiting one or more of the ABCDE criteria, an intra-individual comparative analysis, which involves identifying the lesion that differs from others in the same patient (“ugly duckling” sign) and the assessment of lesion evolution when prior documentation is available. Full-body skin examinations are recommended for early diagnosis and to reduce skin cancer burden, but direct evidence linking skin cancer screening in the general population to improved health outcomes is lacking. Screening asymptomatic individuals may even lead to potential harm, particularly through the overdiagnosis of lesions that would not have caused symptoms if left undetected. Such overdiagnosis can result in unnecessary treatments and impose psychological and social burdens [3,10,23,24].

While the efficiency of routine skin cancer screening continues to be debated, it is widely agreed that dermoscopy should be incorporated whenever such screening is undertaken. According to a meta-analysis conducted on 104 study publications, dermoscopy increases the sensitivity for the detection of melanoma over the naked-eye total-body examination from 76% to 92%, while also improving specificity from 75% to 95% [25].

In addition to dermoscopy, methods like sequential digital mole mapping, potentially linked to 2D or 3D total-body photography, significantly contribute to early melanoma detection, especially in high-risk individuals [26]. Digital mole mapping involves capturing, storing, and comparing dermoscopic images of one or more melanocytic lesions over time and supports the identification of suspicious changes [27].

However, in long-term monitoring of low-risk patients, regular handheld dermoscopic screenings alone appear to be the primary factor associated with reduced Breslow thickness, with no additional benefit from digital mole mapping in terms of Breslow thickness or pT stage distribution [28].

An ongoing objective is therefore to accurately stratify patients according to their individual risk and to develop personalized screening recommendations tailored to their specific needs. This can prevent overdiagnosis and ensure cost-effective care while promoting appropriate, risk-adapted access to dermatological services without overburdening healthcare systems [29].

The primary aim of this retrospective cohort study was to evaluate the impact of dermatologic screening frequency and diagnostic methods on melanoma stage, specifically, Breslow thickness and the proportion of melanoma in situ. We aimed to determine whether different screening strategies are associated with earlier detection of melanoma, particularly in relation to individual risk profiles, and to assess the potential implications for personalized screening recommendations in clinical practice.

## 2. Materials and Methods

We conducted a retrospective cohort study using electronic medical records of all patients with a histopathological diagnosis of cutaneous melanoma between 2017 and 2024 at Erasme Hospital (Brussels, Belgium).

Demographic variables and melanoma characteristics were collected at the time of diagnosis. Quantitative variables (e.g., age) are reported as mean ± standard deviation or median with interquartile range, depending on data distribution. Categorical variables (e.g., sex, skin phototype) are expressed as counts and percentages. Patients with missing data for the respective variables were excluded from the corresponding analyses.

The screening frequency was divided into three categories: (i) irregular screening (a single consultation within the two years prior to melanoma diagnosis); (ii) regular screening (2 to 4 consultations within two years prior to diagnosis); and (iii) close screening (more than 4 consultations within two years prior to diagnosis). 

Participants were further stratified according to their individual melanoma risk. A low-risk group was defined as patients with no personal or family history of melanoma and a total naevus count of ≤100; whereas the high-risk group comprised patients with a personal or family history of melanoma and/or a total naevus count of >100. This stratification was based on meta-analytic evidence demonstrating a strong dose-dependent increase in melanoma risk with higher naevus counts (pooled RR ≈ 6.9 for 101–120 vs. ≤15 naevi), as well as independent risk elevation associated with personal or family history of melanoma [7].

Group comparisons for categorical variables used Pearson’s chi-squared or Fisher’s exact test, with odds ratios reported when significant. Differences in Breslow thickness across screening frequency groups were assessed using the Kruskal–Wallis test, followed by post hoc pairwise comparisons with appropriate *p*-value adjustment. Random-effects logistic regressions assessed the association between follow-up frequency and melanoma in situ diagnosis. Linear mixed-effects models were used to account for repeated measures. Statistical analyses were conducted using Microsoft Excel, JASP (version 0.19.3), and Stata (version 19). A *p*-value < 0.05 was considered statistically significant.

Incidence of in situ and invasive cutaneous melanomas of the Belgian population was obtained by the Belgian Cancer Registry (BCR) for years 2018–2022. The extraction of Breslow thickness from data of the Belgian population (excluding Erasme) was carried out by BCR using pathology protocols [30].

The study was performed in accordance with the Helsinki Declaration of 1964 and its later amendments. Data collection and handling complied with applicable laws, regulations, and guidance regarding patient protection, including patient privacy. The study was approved by the Ethics Committee of Erasme Hospital (Reference: P2024/615).

## 3. Results

### 3.1. Patient Demographics

A total of 475 cutaneous melanomas were diagnosed between 2017 and 2024 in 397 patients (198 females [49.87%] and 199 males [50.13%]). The most represented skin phototype was type II (67%, *n* = 266), followed by type III (20%, *n* = 79) and type I (12%, *n* = 48) (Table 1).

From the 475 diagnosed melanomas, 39.58% (*n* = 188) were in situ and 60.42% (*n* = 287) were invasive. The mean age at diagnosis was 63.75 ± 14.5 years. A first-degree family history of melanoma was reported in 12% of patients (*n* = 46). Among the 397 patients, 18% (*n* = 73) developed two or more melanomas. On average, the time between the first and second melanoma was 5.46 ± 8.27 years. For patients with a third melanoma (*n* = 19), the interval between the second and third was 2.78 ± 4.05 years. For those with more than three melanomas (*n* = 72 melanomas), the average time between subsequent melanomas was 1.54 ± 3.45 years (Table 1).

57.47% (*n* = 273) of all melanomas were identified on first-time dermatological examination at Erasme Hospital. From the remaining melanomas (*n* = 202) identified in patients who were previously followed up, 12.42% (*n* = 59) were diagnosed during irregular screening, 18.74% (*n* = 89) were diagnosed during regular screening and 11.37% (*n* = 54) during close screening.

Patients with multiple melanomas were significantly more frequent in the groups with regular (41.6%, *n* = 37) or close screening (61.1%, *n* = 33) compared to those with no screening at Erasme Hospital (13.6%, *n* = 37) or irregular screening (23.7%, *n* = 14; *p* < 0.001).

### 3.2. Impact of Screening Frequency on Melanoma Stage

The proportion of in situ melanomas, relative to invasive cases, was significantly higher among patients who underwent any form of screening—irregular (*p* = 0.035), regular (*p* = 0.008), or close screening (*p* = 0.046)—compared to those diagnosed at their first consultation. However, the screening frequency within the two years prior to diagnosis was not significantly associated with in situ melanomas: there were no statistically significant differences in the proportion of in situ melanomas within the groups themselves of irregular, regular, or close screening (*p* > 0.05) (Table 2).

The mean Breslow thickness of invasive melanomas in patients without prior screening and diagnosed at their first consultation was 1.8 ± 3.48 mm. In contrast, patients who underwent irregular screening (0.7 ± 0.41 mm), regular (0.68 ± 0.60 mm), or close screenings (0.73 ± 0.53 mm) had significantly lower mean Breslow indices (*p* ≤ 0.001). However, there were no statistically significant differences in Breslow thickness between the groups themselves with irregular, regular, or close screening (*p* > 0.05) (Table 2).

### 3.3. Impact of Screening Method on Melanoma Stage

Full-body skin examination (*n* = 282) was associated with a higher proportion of in situ melanomas compared to targeted lesion screening (46% vs. 34%; *p* = 0.016, respectively). Analysis of invasive cases likewise revealed differences in Breslow thickness according to the extent of the clinical examination: Breslow thickness was significantly lower in those diagnosed through full-body screening compared with patients diagnosed by targeted lesion examination (0.92 ± 1.12 mm vs. 2.05 mm ± 4.48; *p* = 0.02). At the first visit, 70% of melanomas in the targeted lesion group and 38% in the full-body group were diagnosed. Excluding these first-visit cases, the proportion of melanoma in situ was 44% for targeted lesion screening and 51% for full-body examination (*p* = 0.433). For invasive melanomas diagnosed during follow-up, the mean Breslow thickness was 0.67 ± 0.37 mm in the targeted lesion group and 0.69 ± 0.57 mm in the full-body group (*p* = 0.903) (Table 3).

Among the 282 patients who underwent full-body examination, no significant differences in the proportion of in situ melanomas were observed between patients screened by dermoscopy (46% melanoma in situ) and patients receiving digital mole mapping (45%; *p* = 0.995).

However, invasive melanomas diagnosed via digital mole mapping had a lower mean Breslow thickness than those diagnosed with dermoscopy only (0.55 ± 0.28 mm vs. 1.07 ± 1.29 mm; *p* = 0.001).

48% of melanomas in the dermoscopy group and 9% in the digital mole mapping group were diagnosed at the first visit. Excluding these cases, the proportion of melanoma in situ was 53% within the dermoscopy group and 47% within the digital mole mapping cohort (*p* = 0.415). For invasive melanomas detected during follow-up, the mean Breslow thickness was 0.79 ± 0.64 mm with dermoscopy and 0.53 ± 0.28 mm with digital mole mapping (*p* = 0.035) (Table 3).

### 3.4. Screening Frequency and Method: Impacts Stratified by Melanoma Risk

In a second step, participants were stratified according to their individual melanoma risk (as defined in the methods section) to verify whether our former observations were generalizable across the entire melanoma cohort or predominantly driven by specific patient subgroups. There were a total of 142 patients in the low-risk group and 145 patients in the high-risk group. 97 of the high-risk, and 94 of the low-risk patients in both groups had an invasive melanoma and were used for further analysis.

In the low-risk group, median Breslow thickness did not differ significantly across the four screening frequency levels (*p* = 0.292), with medians of 0.645 mm (IQR 0.40–1.10) for patients without any screening in the two years prior to diagnosis, 0.60 mm (IQR 0.40–0.95;) for those with irregular screening, 0.62 mm (IQR 0.51–1.28) for those with regular, and 0.41 mm (IQR 0.40–0.49) for those with close screening (*p* = 0.292) (Table 4).

In the high-risk group, however, a statistically significant association was observed between the median Breslow thickness and the screening frequency (*p* = 0.001). The median Breslow thickness was highest (0.91 mm, IQR 0.48–1.50) in patients without any screening within the two years prior to melanoma diagnosis, while those who underwent irregular (0.55 mm, IQR 0.38–0.922), regular (0.45 mm, IQR 0.30–0.50) or close screening (0.4 mm, IQR 0.00–0.45) screening showed progressively thinner melanomas (Table 4).

We finally analyzed the impact of the screening method on Breslow thickness according to the individual risk level of patients. In the low-risk group, no significant difference in Breslow thickness was observed between melanomas detected by digital mole mapping and dermoscopy (0.56 mm, IQR 0.45–0.64 vs. 0.58 mm, IQR 0.40–0.95; *p* = 0.798).

In contrast, among high-risk patients, digital mole mapping identified melanomas at a significantly lower Breslow thickness compared with dermoscopy alone (0.47 mm vs. 0.6 mm; *p* = 0.020) (Table 4).

### 3.5. Comparison Between the Erasme Hospital Cohort and the Belgian Population

Between 2018 and 2020, the proportion of melanoma in situ at Erasme remained stable (26.5–32.7%), followed by an increase in 2021 (49.4%), 2023 (48.5%), and 2024 (59.6%). In contrast, the national Belgian data showed stable proportions between 2018 and 2022 (32.9–37.5%). The proportion of in situ melanomas was significantly higher at Erasme compared with the national level in 2021 (*p* = 0.024) and in 2024 estimates (*p* = 0.002), but not in other years. After excluding patients without prior follow-up at Erasme, the proportion of in situ melanomas was 55% in 2021 versus 37.5% nationally (*p* = 0.032), and 74.1% in 2024 versus 38.5% nationally (*p* = 0.001), with no significant differences in other years.

Among patients followed at Erasme before diagnosis, invasive melanomas were detected earlier than at the national level (excluding Erasme), with significantly lower Breslow thickness from 2018 to 2022 (range 0.54–0.87 mm vs. 1.48–1.53 mm; all *p* ≤ 0.017) (Table 5).

## 4. Discussion

This retrospective cohort study provides important insights into the influence of skin cancer screening practices—specifically screening frequency and diagnostic method—on the stage of cutaneous melanoma. The findings should be interpreted as associative and exploratory. Our findings show that when analyzing the total melanoma cohort, patients who underwent any dermatological screening within the two years preceding their melanoma diagnosis had significantly thinner tumours and higher melanoma in situ proportions, compared to those diagnosed at their first dermatological consultation. Notably, the mean Breslow index in patients without prior screening was 1.8 mm, whereas it ranged between 0.68 and 0.73 mm in the screened groups. However, within screened patients, increasing screening frequency was not associated with a higher proportion of in situ melanomas, nor with a lower Breslow thickness (*p* > 0.05).

These findings align with a study from the U.S. among 595,799 individuals, showing that 144,851 (24.3%) patients who were (ever) screened were more likely than unscreened (never) patients to be diagnosed with in situ (HR 2.6; 95% CI 2.1–3.1; *p* < 0.001) or thin invasive (≤1 mm) melanoma (HR 1.8; 95% CI, 1.5–2.2; *p* < 0.001), compared to those that were eligible for screening but did not take part [31]. However, this study did not account for the temporal delay between the last screening visit and melanoma diagnosis. The absence of this information limits interpretation. Studying the impact of periodical screenings, Morr et al. demonstrated that patients screened at intervals of 12 months or less prior to their diagnosis have thinner melanomas at presentation than their counterparts who had never had skin cancer screening or who had an interval of over 12 months prior to a melanoma diagnosis [32].

When focusing not on tumour thickness, but on mortality rate, similar trends have been observed: Screening participants were not only diagnosed at lower UICC/AJCC stages (approximated by the lower proportion of locoregional or distant metastases within 100 days of initial diagnosis) but also more favourable outcomes compared to those who had not undergone any dermatological screening within the two years prior to their melanoma diagnosis. Specifically, screening participants demonstrated a significantly higher overall survival rate and reduced mortality risk (HR 0.62, 95% CI 0.48–0.80) [22]. A major methodological limitation of the study is that it assessed overall survival, but not melanoma-specific survival or the risk of death directly attributable to melanoma. Consequently, it remains uncertain whether the observed mortality benefit is truly attributable to earlier diagnosis and better prognosis of melanoma. The effect could just as well reflect generally healthier behaviour among screening participants (e.g., greater use of preventive services, fewer comorbidities) or other causes of death unrelated to melanoma. Thus, when interpreting the reported reductions in Breslow thickness and excess mortality among screening participants, both the “healthy screened bias” and potential overdiagnosis associated with screening need to be considered [33,34,35,36]. In fact, population-level data from the United States (2010–2016) show a continuous rise in melanoma incidence without a corresponding decline in melanoma-specific mortality, a pattern interpreted as evidence of overdiagnosis. Stable rates of metastatic melanoma further support the notion that the true occurrence of clinically relevant disease has not increased substantially. This epidemiologic signature illustrates that greater diagnostic scrutiny may inflate case numbers while offering limited impact on melanoma-specific mortality. Besides effectiveness, screening programmes must also demonstrate efficient use of resources. The estimated number needed to screen (NNS) to prevent one death among melanoma patients is estimated at 8321 (95% CI 7561–9249), which represents a remarkably high figure for a population-based screening programme [22,31,37].

This raises important questions about how skin cancer screening can be organized in a more targeted and resource-efficient manner. One possible approach is lesion-directed screening strategies, such as the recently implemented “one spot check” consultations of the Dermatology Department of Ghent University Hospital, which have demonstrated high detection rates while avoiding the extensive costs of population-wide screening [38,39]. Lesion-directed screening through early-access dermatology consultations has shown to be an efficient alternative to total-body examination in the general population. In an observational study, patients presenting with one or two predefined suspicious lesions achieved a skin cancer detection rate of 13.2% (4.1% for melanoma), with even higher yields in referred patients or those with a history of skin cancer [38]. In addition to surveillance programmes for high-risk patients, lesion-directed screening may help optimize skin cancer detection in the general population while allowing for a more efficient use of time in daily dermatology practice.

Our analysis demonstrated that full-body skin examinations were more frequently associated with the diagnosis of in situ melanomas (46% vs. 34%; *p* = 0.016) and with lower Breslow thickness (0.92 mm ± 1.12 vs. 2.05 mm ± 4.48; *p* = 0.02), compared to targeted lesion assessments. This suggests that, while lesion-directed strategies can optimize efficiency and maintain high detection rates, systematic full-body examination may contribute added value by enabling the identification of very early-stage melanomas that might otherwise remain undetected. When restricting the analysis to follow-up patients, the observed advantage of full-body examination over lesion-directed screening with respect to the proportion of in situ melanomas and mean Breslow thickness was no longer statistically significant. The differences in the overall cohort could be confounded by referral bias: externally referred, first-time patients are more likely to present with clinically evident or more advanced melanomas compared to follow-up patients.

Since total body examination in the general population is not cost-effective, one possible approach could be to perform a total-body examination specifically in high-risk patients and individuals presenting with a suspicious index lesion during a dermatological examination: data from “one-spot-consultations” of the Dermatology Department of Ghent University Hospital performing a subsequent total-body examination in patients with a clinically benign index lesion resulted in a low additional detection rate (0.5%), whereas in patients with a suspicious index lesion, an additional detection rate of 8.9% was achieved. These data suggest that total body examination following the lesion-directed screening approach is strongly advisable in cases of a suspicious index lesion but may be less (cost-) effective when the index lesion is benign [38].

Having compared the extent of examination, we next evaluated the diagnostic technology applied. Among patients who underwent a full-body examination, the proportion of in situ melanomas was nearly identical between dermoscopy and digital mole mapping. However, when focusing on invasive melanoma cases, digital mole mapping was associated with a markedly lower mean Breslow thickness (0.55 mm), whereas standard dermoscopy alone yielded significantly thicker melanomas (1.07 mm) (*p* = 0.001).

In the comparison of dermoscopy and digital mole mapping, exclusion of first-visit patients revealed once again no significant difference in the proportion of melanoma in situ between the two modalities. However, digital mole mapping still identified invasive melanomas with a significantly lower mean Breslow thickness compared to dermoscopy. This suggests that the advantage of digital mole mapping in detecting thinner tumours is not merely explained by first-visit bias but reflects a methodological benefit in the context of longitudinal follow-up.

Our data indicate that any screening within 2 years prior to melanoma diagnosis is associated with thinner tumours and a higher proportion of in situ melanomas, yet increasing screening frequency did not translate into further improvements. But the overall number needed to screen to avoid one melanoma death remains high (8.321), and the efficiency of screening programmes could potentially be improved by targeting high-risk groups [22]. As our main analysis did not adjust for individual risk, we performed a risk-based subgroup analysis in the next step: In the low-risk group, no statistically significant association between screening and tumour thickness was observed. Nevertheless, a tendency towards thinner melanomas with increasing screening frequency was evident. No difference was observed regarding the diagnostic modality: melanomas detected by digital mole mapping showed comparable tumour thickness to those diagnosed by dermoscopy. With larger case numbers, these differences might potentially reach statistical significance; however, given the already very high number needed to screen, the clinical and public health relevance of such a finding in low-risk patients appears questionable.

A study of 621 melanomas in low-risk patients (<50 naevi, no personal or family history) aligns with our findings in showing that periodical digital mole mapping does not significantly differ from handheld dermoscopy regarding pT stage distribution, mean Breslow thickness, or ulceration in low-risk individuals. However, they reported that regular dermoscopic examinations enabled significantly earlier melanoma detection compared to first-time assessments, even in this low-risk group [28].

In the high-risk group, a statistically significant association between screening and melanoma stage was observed: individuals without prior screenings had the greatest tumour thickness (0.905 mm), while it decreased progressively (0.55–0.4 mm) with an increasing number of screening visits. These findings are in accordance with the actual European Guideline, stating that sequential 2D or 3D whole-body photography and digital dermoscopy significantly contribute to early melanoma detection in the context of high-risk individuals. Known high-risk groups (genetic predisposition, personal melanoma history, high total nevus count, etc.) are likely to benefit from whole-body photography [3,26]. Sequential dermatoscopic documentation is especially valuable in high-risk individuals with multiple atypical moles, facilitating both the early detection of melanoma and minimizing excisions of benign lesions [40,41,42].

Several studies have examined the effectiveness of skin cancer screening in individuals at elevated risk for melanoma. A study from Sweden (1987–2001) demonstrated that a coordinated programme for early detection and skin surveillance in families with hereditary cutaneous melanoma was associated with favourable histopathologic features, with a median tumour thickness of 0.5 mm and absence of ulceration in most cases (92%) [43]. Another study in melanoma-prone families showed that participation in a structured longitudinal surveillance program that included regular skin cancer screening and education on skin self-examinations was associated with the detection of significantly thinner melanomas [44]. In the prospective cohort, tumours were markedly thinner than pre-study cases (0.6 mm vs. 1.1 mm) and more often diagnosed at stage T1 (83% vs. 40%) [44]. These findings underscore the clinical value of physician-led and self-examination screening in high-risk individuals.

Structured screening programmes have been shown to improve early detection and lead to more favourable tumour characteristics in high-risk individuals, shifting attention toward optimizing methods and efficiency. Digital techniques such as mole mapping and total body photography have already demonstrated value by increasing surveillance sensitivity while limiting unnecessary excisions. The next focus is on risk prediction models, which aim to enhance efficiency further by lowering the number needed to screen through risk-adapted strategies. These represent a promising tool for improving the efficiency of melanoma detection at the population level. The underlying principle is to target individuals at greatest risk with skin examinations, while minimizing low-yield screening among those at lower risk. To date, more than 40 predictive models for individual melanoma risk have been developed. A recent Australian risk prediction model (MP16) indicated that targeting the top 40% of the population by predicted risk would capture 74% of invasive melanoma cases (NNS = 32). Expanding to the top 50% increased case capture to 82%, with an NNS of 36. Decision curve analysis demonstrated a net benefit compared with universal or no screening [29]. The model was developed within the QSkin cohort and is based on 16 predictors. These include age (with a quadratic term), sex, and an age-by-sex interaction term, as well as ancestry (European vs. other), nevus density, freckling density, hair colour, tanning ability, number of adult sunburns, family history of melanoma, prior cancer, previous skin cancer excisions, history of treated actinic keratoses, smoking status, and height. Compared with other risk stratification models, the MP16 model offers superior accuracy and reduced bias by integrating more risk factors and robust validation in a large, population-based cohort.

## 5. Conclusions

In conclusion, this retrospective cohort study demonstrates that dermatological screening within two years prior to melanoma diagnosis is associated with thinner tumours and a higher proportion of in situ diagnoses. While increasing screening frequency beyond regular intervals did not yield additional benefit in the overall cohort and screening was not shown to be effective in low-risk patients, high-risk patients showed a progressive reduction in Breslow thickness with more frequent follow-up visits. Furthermore, digital mole mapping proved superior to dermoscopy in detecting thinner invasive melanomas, even after excluding first-visit cases, and this advantage was particularly evident in high-risk patients, underlining its specific value in longitudinal monitoring of individuals at increased risk. These findings support a risk-adapted approach in which surveillance and advanced diagnostic modalities are concentrated on high-risk patients, while lower-risk individuals may be adequately managed with less resource-intensive strategies. Future prospective studies are needed to validate these observations and further optimize personalized melanoma screening algorithms.

## Figures and Tables

**Table 1:** Demographics. This table summarizes the study cohort by sex, skin phototype (I–III), first-degree family history, multiplicity of melanomas, and intervals between successive melanomas. Values are reported as counts and percentages or as mean ± standard deviation, as appropriate. Abbreviations: *n* = number; % = percent.

Variable	Value
Melanomas, *n* (patients, *n*)	475 (397)
Female, *n* (%)	198 (49.87)
Male, *n* (%)	199 (50.13)
Skin phototype I/II/III, *n* (%)	48 (12.1)/266 (67.0)/79 (19.9)
First-degree family history of melanoma, *n* (%)	46 (12.0)
≥2 melanomas, *n* (%)	73 (18.4)
Interval 1st → 2nd melanoma, years	5.46 ± 8.27
Interval 2nd → 3rd melanoma, years	2.78 ± 4.05
Interval between subsequent (>3) melanomas, years	1.54 ± 3.45

**Table 2:** Impact of screening frequency within two years prior to diagnosis on melanoma stage. Shown are proportions of melanoma in situ and mean Breslow thickness according to screening frequency in the two years before diagnosis: first visit (no prior screening at Erasme), Irregular (1 visit), Regular (2–4 visits), and Close screening (>4 visits). Odds ratios are calculated versus first visit (reference). Abbreviations: *n* = number; OR = odds ratio; Ref. = reference category; mm = millimetres; SD = standard deviation; *p* = *p*-value.

Screening	*n*	In Situ, % (*n*)	OR vs. First Visit	*p* (In Situ)	Breslow (mm), ± SD	*n*	*p* (Breslow)
First-visit	273	32.6 (89)	Ref.	—	1.80 ± 3.48	184	—
Irregular	59	50.9 (30)	2.42	0.035	0.70 ± 0.41	29	≤0.001
Regular	89	50.6 (45)	2.55	0.008	0.68 ± 0.60	44	≤0.001
Close	54	44.4 (24)	2.05	0.046	0.73 ± 0.53	30	≤0.001

**Table 3:** Impact of diagnostic method and extent of examination on melanoma stage. This table compares examination extent (Full-body exam vs. Lesion-directed) and diagnostic method (Dermoscopy vs. Mole mapping) for the full cohort and, separately, for cases diagnosed during longitudinal care (Follow-up only, excluding first visits). Reported are the proportion of melanoma in situ and the mean Breslow thickness. Abbreviations: *n* total = total number; Follow-up only = cases diagnosed during longitudinal follow-up (first visit excluded); mm = millimetres; SD = standard deviation.

	Method	*n* Total	In Situ, % (*n*)	Breslow (mm), Mean ± SD	*n*	*p*-Value
Full cohort	Full-body exam	282	46 (129)	0.92 ± 1.12	153	0.016 (in situ); 0.020 (Breslow)
	Lesion-directed	143	34 (48)	2.05 ± 4.48	95	
Follow-up only	Full-body exam	175	51 (89)	0.69 ± 0.57	86	0.433 (in situ); 0.903 (Breslow)
	Lesion-directed	43	44 (19)	0.67 ± 0.37	24	
Full cohort	Dermoscopy	212	46 (98)	1.07 ± 1.29	115	0.995 (in situ); 0.001 (Breslow)
	Mole mapping	64	45 (29)	0.55 ± 0.28	35	
Follow-up only	Dermoscopy	111	53 (59)	0.79 ± 0.64	52	0.415 (in situ); 0.035 (Breslow)
	Mole mapping	58	47 (27)	0.53 ± 0.28	31	

**Table 4:** Risk-stratified effects of screening frequency and method on Breslow thickness. Median Breslow thickness with interquartile range (IQR) is presented for each risk group (Low-risk and High-risk) by screening frequency or diagnostic method. Abbreviations: *n* = number; IQR = interquartile range; mm = millimetres; Ref. = reference category.

Risk Group		Screening	*n*	Breslow (mm) [IQR]	*p*-Value
Low-risk Group (*n* = 94)	Frequency	First visit	68	0.65 [0.40–1.10]	Ref.
		Irregular	11	0.60 [0.40–0.95]	
		Regular	11	0.62 [0.51–1.28]	
		Close	7	0.41 [0.40–0.49]	0.292
	Method	Dermoscopy	79	0.58 [0.40–0.95]	Ref.
		Mole mapping	4	0.56 [0.45–0.64]	0.798
High-risk Group (*n* = 97)	Frequency	First visit	42	0.91 [0.48–1.50]	Ref.
		Irregular	12	0.55 [0.38–0.92]	
		Regular	17	0.45 [0.30–0.50]	
		Close	3	0.40 [0.00–0.45]	0.001
	Method	Dermoscopy	47	0.60 [0.40–1.25]	Ref.
		Mole mapping	18	0.47 [0.31–0.63]	0.020

**Table 5:** Breslow thickness among followed patients (Erasme) versus the Belgian population, 2018–2022. Year-by-year comparison of mean Breslow thickness (with 95% confidence intervals) between patients followed at Erasme prior to diagnosis and the Belgian comparator population (Belgian Cancer Registry, Erasme excluded). Abbreviations: *n* = number; mm = millimetres; 95% CI = 95% confidence interval; excl. = excluding.

Year	Erasme (Followed Patients, *n*)	Breslow (mm) ± 95% CI	Belgium (Excl. Erasme, *n*)	Breslow (mm) ± 95% CI	*p*-Value
2018	8	0.87 ± 0.48	3128	1.51 ± 0.11	0.017
2019	13	0.54 ± 0.21	3365	1.48 ± 0.08	<0.001
2020	21	0.65 ± 0.14	3244	1.52 ± 0.12	<0.001
2021	18	0.84 ± 0.39	3575	1.48 ± 0.10	0.003
2022	17	0.68 ± 0.31	3721	1.53 ± 0.09	<0.001

## Data Availability

The data supporting the findings of this study are not publicly available due to ethical and privacy restrictions related to patient medical records.
